# Life-Threatening Diffuse Alveolar Hemorrhage as an Initial Presentation of Microscopic Polyangiitis: COVID-19 as a Likely Culprit

**DOI:** 10.7759/cureus.14403

**Published:** 2021-04-10

**Authors:** Rutwik Patel, Viralkumar Amrutiya, Moaaz Baghal, Manan Shah, Abraham Lo

**Affiliations:** 1 Internal Medicine, Hackensack Meridian Health Palisades Medical Center, North Bergen, USA

**Keywords:** diffuse alveolar hemorrhage, microscopic polyangiitis, covid-19, anti-neutrophil cytoplasmic antibody, vasculitis

## Abstract

Microscopic polyangiitis (MPA) is a systemic small vessel vasculitis but it is rare to see life-threatening diffuse alveolar hemorrhage (DAH) in MPA as an initial presentation. MPA more commonly presents with renal involvement and develops pulmonary manifestations later in the disease course. Our patient is a 77-year-old female with a recent history of recovered COVID-19 infection who presented with sudden onset fever, dyspnea, and hemoptysis for three days. She was diagnosed with MPA because of the new-onset DAH, a strongly positive myeloperoxidase (MPO) antibody, and the low likelihood of another etiology. The patient was treated with pulse-dose steroids and plasmapheresis while being on mechanical ventilation. This case highlights the importance of the prompt recognition of DAH as an initial presentation of MPA and illustrates the possible role of COVID-19 in inciting autoimmune conditions.

## Introduction

Microscopic polyangiitis (MPA) is characterized by a systemic, pauci-immune, and necrotizing small-vessel vasculitis, which is a type of anti-neutrophil cytoplasmic antibody (ANCA) vasculitis [1,2]. It is differentiated from other forms of ANCA vasculitis by the absence of clinical or pathological evidence of granulomatous inflammation. Typical initial symptoms include fever, malaise, anorexia, and weight loss for weeks to months, without specific organ involvement [1,3]. Systemic complications like diffuse alveolar hemorrhage and rapidly progressive glomerulonephritis (RPGN) are relatively common as a late presentation. However, here, we report a rare case of MPA with pulmonary-renal syndrome as the initial presentation [2,4]. The patient was also COVID-19 immunoglobulin G positive (COVID-19 IgG) with a negative polymerase chain reaction (PCR) nasal swab. Cytokine storm induced by COVID-19 could be a possible triggering event for expression of myeloperoxidase-ANCA (MPO-ANCA) and neutrophil activation which leads to MPA manifestations [5,6].

## Case presentation

**Figure 1:**
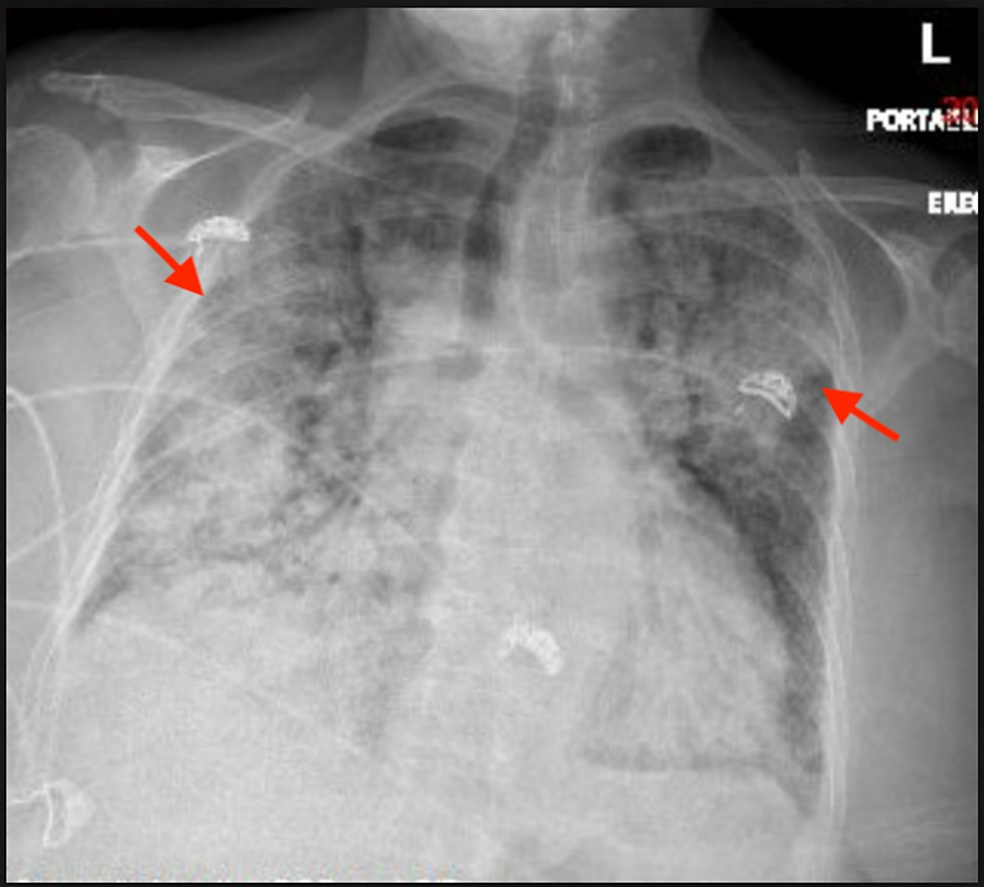
Frontal chest X-ray on admission showed severe scattered patchy bilateral lung infiltrates, diffusely in the right lung and in the left upper lobe (red arrows).

**Figure 2:**
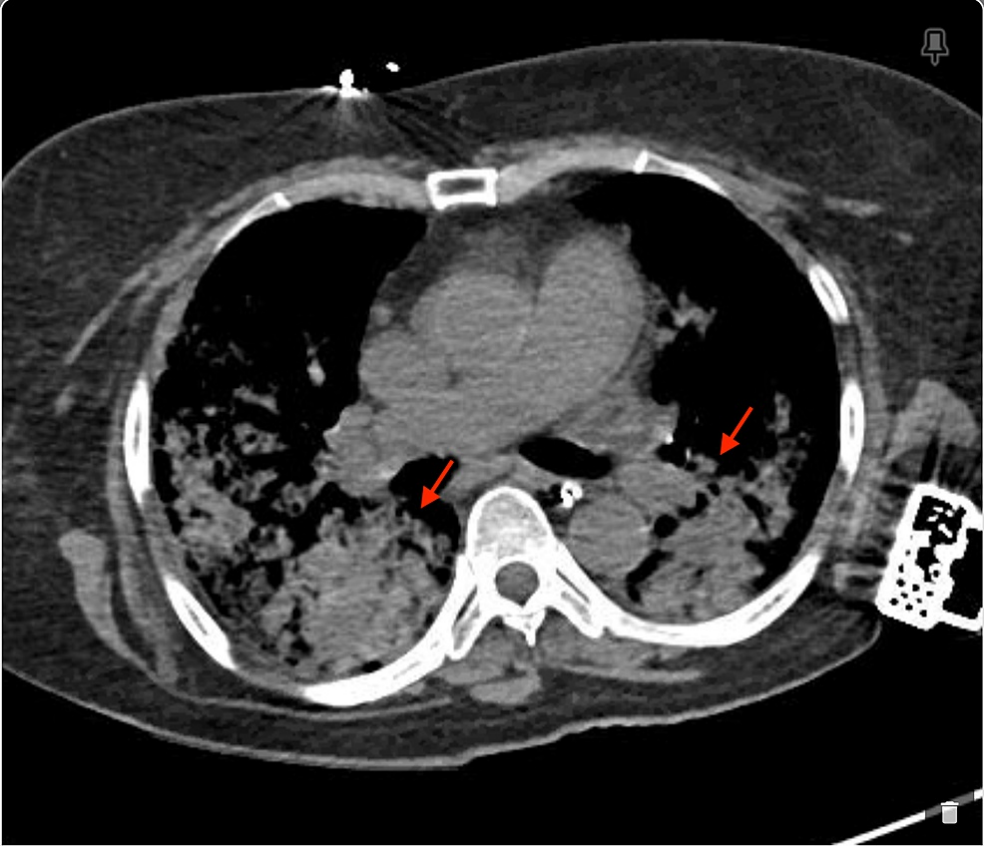
Computerized tomography of the chest without contrast, axial view on admission showed severe, diffuse, bilateral lung disease, consolidative and ground-glass appearance (red arrows), no effusion, mild reactive lymphadenopathy.

**Figure 3:**
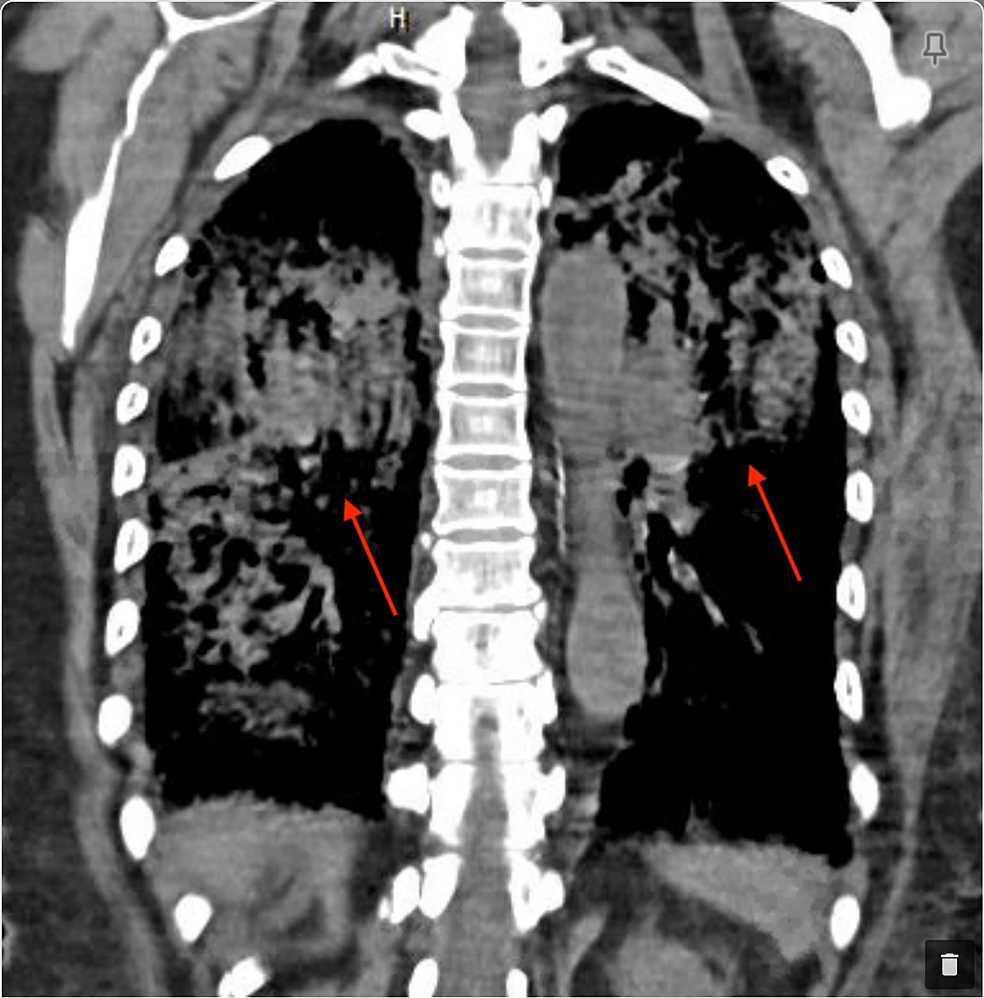
Computerized tomography of the chest without contrast, sagittal view on admission showed severe, diffuse, bilateral lung disease, consolidative and ground-glass appearance (red arrows), no effusion, mild reactive lymphadenopathy.

A 77-year-old female with hypertension, hyperlipidemia, and diabetes mellitus initially presented with fever, dyspnea, and hemoptysis for three days. She was found to have a negative COVID-19 PCR but IgG antibody positive with significantly elevated creatinine. Of note, the patient had mild upper respiratory tract symptoms from COVID-19 infection about six weeks ago diagnosed with PCR test, which had resolved with only symptomatic treatment. On admission, the patient was in mild respiratory distress and a chest X-ray showed bilateral infiltrates as shown in Figure 1. Subsequently, she developed ventilator-dependent respiratory failure and was started on pulse dose steroids with a preliminary diagnosis of DAH. This was confirmed with a chest CT showing diffuse bilateral ground-glass, consolidative appearance, and mild bronchiectasis suggestive of possible alveolar hemorrhage as shown in Figures 2 and 3, as well as bronchoscopy. Bronchoalveolar lavage cytology showed degenerating bronchial cells, abundant alveolar macrophages, fibrous material, negative for malignant cells. Echocardiography showed a left ventricular ejection fraction of 55-60% and normal left ventricular size without any significant valvular abnormalities.

Extensive serologic workup to rule out vasculitis showed MPO antibody positivity 179.4 antibody unit (AU)/milliliter (ml), anti-glomerular basement membrane antibody negative, antinuclear antibody positive with titer 1:40 and homogenous pattern, anti-double-stranded deoxyribonucleic acid negative, rheumatoid factor negative, complement 3 slightly low at 68 mg/dl and complement 4 normal at 16.5 mg/dl. Further workup for infectious etiology including respiratory viral panel, human immunodeficiency virus, mycoplasma pneumonia, pertussis, chlamydia pneumonia, and Quantiferon gold plus was negative but severe acute respiratory syndrome coronavirus 2 immunoglobulin G antibody was positive. In the presence of a new-onset DAH, strongly positive MPO antibody, and low likelihood of another etiology, a presumptive diagnosis of MPA was made.

**Figure 4:**
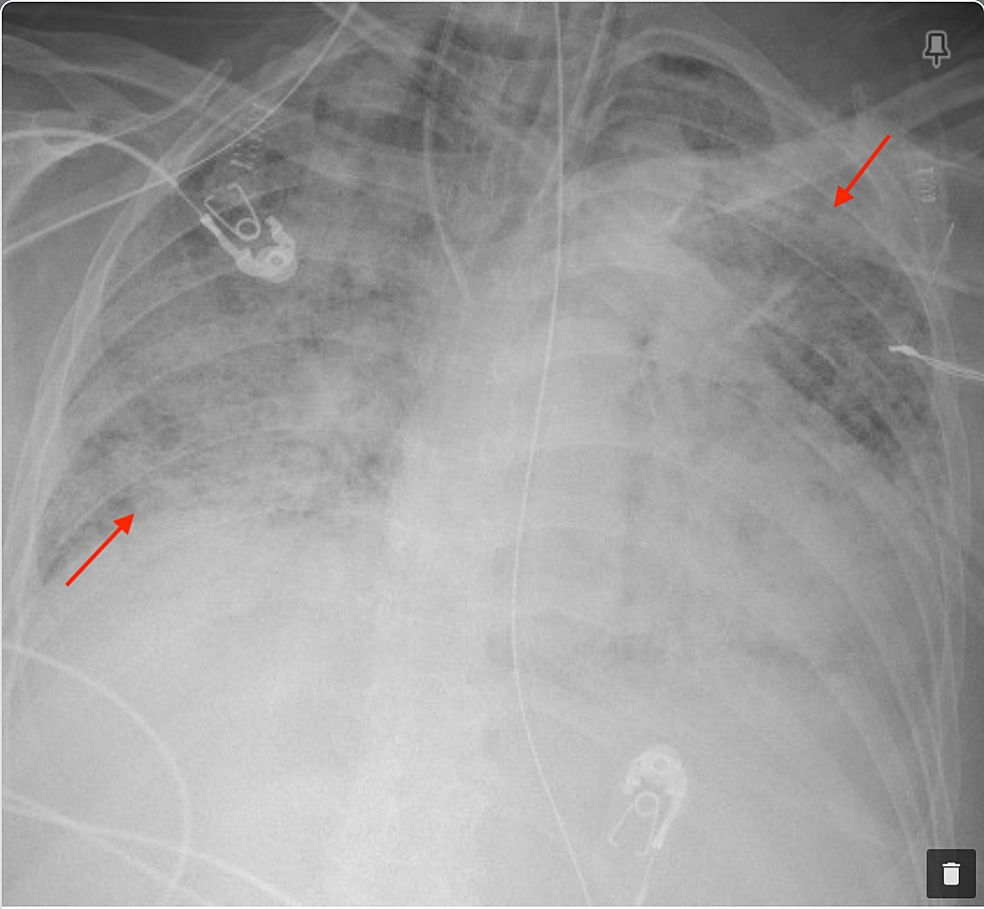
Frontal chest X-ray during hospitalization before starting plasmapheresis showed diffuse alveolar infiltrates bilaterally progressed from prior study compatible with worsening of alveolar hemorrhage or pulmonary edema

**Figure 5:**
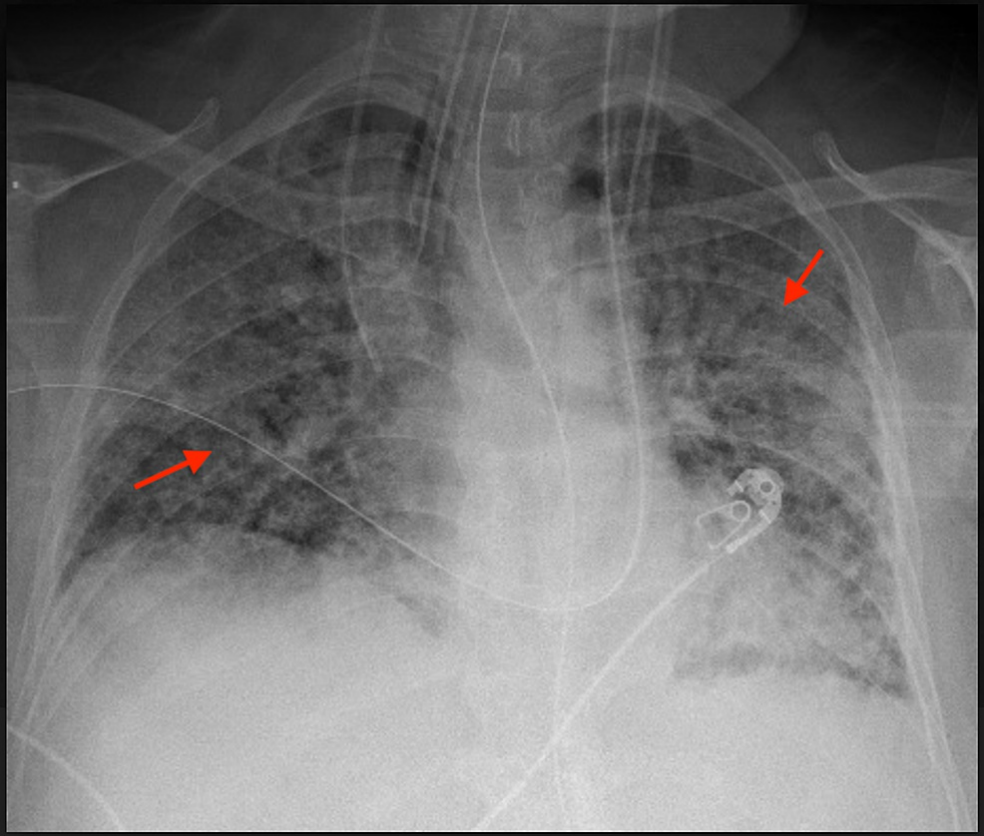
Frontal chest X-ray showing improving diffuse bilateral alveolar infiltrates (red arrows) compared to prior images after initiation of treatment

The patient was transferred to a tertiary care center for plasmapheresis. At that point, a chest X-ray revealed worsening of diffuse alveolar infiltrate as shown in Figure 4. After ruling out infections, she was placed on cyclophosphamide with simultaneous plasmapheresis. As shown in Figure 5, she had improved diffuse bilateral infiltrates initially and her arterial blood gas analysis depicted a trend of improving oxygenation requiring less positive end-expiratory pressure and a fraction of inspired oxygen. The patient had a tracheostomy and peg tube placed and was receiving dialysis every other day at the tertiary care center. She received 10 sessions of plasmapheresis uneventful. Unfortunately, due to hypoxic brain injury, the patient failed spontaneous breathing trials. After the goals of care discussion with the patient’s daughter, the decision was made to pursue comfort care.

## Discussion

MPA is a type of ANCA vasculitis, which is characterized by necrotizing vasculitis involving the small vessels without granulomatous inflammation [1,2]. According to Chapel Hill Consensus Conference criteria, ANCA vasculitis includes granulomatosis with polyangiitis (GPA), eosinophilic granulomatosis with polyangiitis (EGPA), and MPA, in which MPA is distinguished by the absence of granuloma formation [2,3]. Even though the diagnosis by tissue histology is preferred, the European Medicines Agency algorithm suggested criteria for diagnosing ANCA vasculitis where biopsy is not feasible [4,7]. These criteria include the presence of lower and upper airway involvement along with glomerulonephritis in the presence of ANCA [3,7]. In our case, acute hypoxic respiratory failure precluded the kidney biopsy, but the presence of strongly positive MPO-ANCA, radiographic, and bronchoscopic evidence of DAH alluded to the diagnosis of MPA. Favorable clinical response to intravenous steroids and plasmapheresis also supported the diagnosis of MPA [7].

The etiology of MPA remains unclear, but some researchers have attributed genetic, environmental, and immunologic factors such as ANCA to the pathogenesis of MPA [3,8]. One hypothesis explains it in two-step phenomena. As a first step, neutrophils are primed by exposure to proinflammatory cytokines, such as interleukin-1 or tumor necrosis factor-α, which in turn leads to surface expression of MPO-ANCA, followed by adherence of neutrophils to the endothelial surface of blood vessels or glomeruli [9]. In the second step, neutrophils are activated by interaction with MPO-ANCA, through interaction with neutrophil Fc receptors. ANCAs also increase neutrophil adherence to endothelial cells, and the coexistence of ANCA-activated neutrophils and endothelial cells results in endothelial cell lysis [10]. The concept that has emerged from the in vivo studies is that ANCAs and pro-inflammatory stimuli, such as a viral infection, synergize to cause a full-blown inflammatory process [10]. COVID-19 has been implicated to cause a cytokine storm through the release of IL-1, IL-6, TNFα, etc. [4]. COVID-19 infection might have acted as a triggering event for ANCA vasculitis in this patient to initiate a destructive inflammatory process manifesting as a pulmonary-renal syndrome.

MPA often presents with a long prodromal phase of constitutional symptoms such as fever, malaise, and weight loss as well as arthralgias followed by the development of RPGN. Glomerulonephritis is essentially a universal finding in MPA, with pulmonary involvement seen in only up to 30% of patients as a late clinical sign [4]. Pulmonary manifestations include pulmonary fibrosis and pulmonary hypertension, whereas DAH is a lethal complication of an MPA flare. Chest radiographic findings of DAH consist of ground-glass opacity, consolidation, thickened bronchovascular bundles, and a honeycomb appearance caused by alveolar hemorrhage [11,12]. 

The mainstay of therapy for patients with alveolar hemorrhage due to MPA includes aggressive immunosuppression and plasma exchange [8,13]. In organ threatening cases, mechanical ventilation is warranted to maintain oxygenation. In such cases, the treatment plan has two main components: induction of remission with initial immunosuppressive therapy and maintenance of remission with immunosuppressive therapy for a longer duration to prevent relapse. Initial immunosuppression is achieved by high-dose glucocorticoids combined with either cyclophosphamide or rituximab, while the role of plasma exchange remains controversial but is universally performed [13,14]. Our patient initially had significant improvement in lung lesions with steroids and cyclophosphamide along with plasmapheresis. However, due to her late presentation with life-threatening DAH and prolonged hypoxic respiratory failure, she was unable to regain mental capacity and the decision was made to withdraw care.

## Conclusions

In conclusion, novel coronavirus is a new entity in the medical world, which has been implicated in vast numbers of pathologies involving multiple organs. Emerging studies indicate a possible link between COVID-19 and the subsequent onset of autoimmune conditions as a sequela. Association between MPA and COVID-19 has not been established in the current literature. Therefore, the role of COVID-19 in the induction of MPA-like life-threatening disease and other autoimmune diseases remains a topic of further research.
